# Coexistence of Alport Syndrome and Fabry Disease in a Female with R112H Variant: Early Progression of Fabry Nephropathy

**DOI:** 10.3390/ijms27010269

**Published:** 2025-12-26

**Authors:** Amedeo Grimaldi, Alessandra Auletta, Francesca Ciurli, Valeria Aiello, Gisella Vischini, Benedetta Fabbrizio, Francesca Becherucci, Gianandrea Pasquinelli, Gaetano La Manna, Irene Capelli, Renzo Mignani

**Affiliations:** 1 Department of Medical and Surgical Sciences (DIMEC), Alma Mater Studiorum—University of Bologna, 40126 Bologna, Italy; alessandra.auletta@studio.unibo.it (A.A.);; 2 Nephrology, Dialysis and Kidney Transplant Unit, IRCCS Azienda Ospedaliero-Universitaria di Bologna, 40126 Bologna, Italy; 3 Pathology Unit, IRCCS Azienda Ospedaliero-Universitaria di Bologna, 40126 Bologna, Italygianandr.pasquinelli@unibo.it (G.P.); 4 Nephrology and Dialysis, Meyer Children’s Hospital IRCCS, 50139 Florence, Italy; 5 Department of Biomedical, Experimental and Clinical Sciences, University of Florence, 50121 Florence, Italy

**Keywords:** Fabry disease, Alport syndrome, *p.R112H* variant, *COL4A4* variant, chronic kidney disease

## Abstract

Fabry disease (FD) is an X-linked lysosomal disorder caused by *GLA* mutations, typically associated with glycosphingolipid accumulation and a wide phenotypic spectrum. The *p.R112H* variant is generally linked to a non-classic predominantly renal phenotype with mild biochemical abnormalities and slow progression. We report the case of a young woman carrying the *R112H* mutation who exhibited early-onset kidney involvement and unusually rapid progression to end-stage renal disease. Clinical history, serial evaluations, and kidney biopsy findings initially supported a diagnosis of Fabry nephropathy; however, re-evaluation of the native kidney biopsy revealed marked remodeling and multilamellation of the glomerular basement membrane, suggesting Alport-like lesions. Subsequent genetic testing confirmed a heterozygous pathogenic *COL4A4* variant (*G912R*), indicating coexistence of Fabry disease and autosomal dominant Alport syndrome. This dual genetic condition likely accounted for the accelerated decline in kidney function, in contrast with the typically mild phenotype associated with *R112H*. Our literature review indicates that coexistence of these two inherited nephropathies has not previously been confirmed either histologically or genetically. This case underscores the importance of integrating genetic and ultrastructural assessment in patients with atypical or rapidly progressive renal disease

## 1. Introduction

Fabry disease (FD, OMIM 301500) is a rare inherited storage disorder caused by mutations in the *GLA* gene (located at Xq22) that result in deficient or absent lysosomal α-galactosidase A (*GLA*) activity [1]. The ubiquitous presence of lysosomes, essential for breaking down and recycling cellular materials, suggests that FD has a broad impact, influencing various organs and exhibiting a range of phenotypes shaped by the degree of their involvement [2]. Enzymatic defects lead to an accumulation of glycosphingolipids, such as globotriaosylceramide (Gb3) and globotriaosylsphingosine (lyso-Gb3), in multiple cell types, tissues, and organs [3]. It has been demonstrated that exposure to plasma lyso-Gb3 correlates with the severity of disease manifestations [4].

The clinical presentation of FD is highly variable, with phenotypes ranging from the early onset “classic form” (no residual enzymatic activity, markedly elevated lyso-Gb3 levels, and rapid progression to organ failure) to the “late-onset form” (delayed onset disease with low residual enzymatic activity, mildly elevated lyso-Gb3 levels, and slow progression).

In patients with classic form, symptoms typically appear in childhood and involve multiple organs with acroparesthesia at the extremities, angiokeratomas, hypohidrosis, cornea verticillata, gastrointestinal symptoms (such as abdominal pain or diarrhea), and albuminuria. As patients progress into adolescence and adulthood, they may develop overt proteinuria and chronic renal failure, hypertrophic cardiomyopathy, and cerebrovascular complications. Kidney function progressively declines, often leading to end-stage kidney disease (ESKD) in males in the third to fifth decade.

By contrast, in late-onset phenotypes, patients exhibit residual leukocyte α-Gal A activity ranging from 10% to 30%, symptom onset typically occurs in the fourth to fifth decades of life [5], while disease progression and severity are variable, with symptoms predominantly involving cardiac complications.

Heterozygous females may either remain asymptomatic or experience milder symptoms with a later onset compared to males. However, it is not uncommon for some females to present with symptoms as severe as those seen in males with the classic phenotype. This phenotypic variability among heterozygotes can be influenced by several factors, including random X-chromosome inactivation [6].

More than 1000 different *GLA* variants have been identified in patients with Fabry disease (http://fabry-database.org (accessed on 20 November 2025). Most *GLA* mutations are linked to a non-classic phenotype; however, in a significant number of cases, the diagnosis of Fabry disease remains uncertain due to the presence of genetic variants of unknown significance (VUS) [7].

The *p.R112H* (*c.335G > A*) variant is generally associated with a late-onset phenotype, and it is characterized by almost normal to mildly elevated lyso-Gb3 levels and mild clinical symptoms, despite relatively low *GLA* enzymatic activity. It is thought to involve only the renal system and generally occurs in the advanced decades of life [8,9,10,11].

Alport syndrome (AS) is an inherited disorder caused by mutations in the *COL4A5* (OMIM 303630), *COL4A4* (OMIM 120131), and *COL4A3* (OMIM 120070) genes, which compromise the integrity of the type IV collagen network within the glomerular basement membrane (GBM), leading to X-linked, autosomal, or digenic patterns of inheritance [12,13].

The X-linked form (XLAS), resulting from *COL4A5* mutations, represents the most common type and accounts for approximately 80% of all Alport syndrome cases [14].

In contrast, the autosomal dominant (ADAS) and autosomal recessive (ARAS) forms are estimated to account for about 5% and 15% of cases, respectively [15].

Recent evidence suggests that pathogenic variants in *COL4A3* and *COL4A4* may be more frequent than previously recognized [16].

Clinically, the disease presents with hematuria, proteinuria, and progressive renal failure, often accompanied by hearing impairment and ocular defects [17].

In this study, we report the case of a young woman with Fabry disease caused by the *GLA R112H* variant, characterized by early kidney involvement that rapidly progressed to end-stage kidney disease (ESKD), in whom renal biopsy revealed Alport-like lesions, further confirmed by the identification of a heterozygous *COL4A4* mutation (*G912R*).

We reviewed the literature, analyzed the clinical characteristics and pathological findings, and shared pathophysiological mechanisms between Fabry disease and Alport syndrome, aiming to provide clinical insights and facilitate more accurate diagnoses. To the best of our knowledge, this is the first histologically proven case reporting the coexistence of these two genetic diseases.

## 2. Case Description

A 40-year-old woman (weight 62 kg, height 167 cm, BMI 21 Kg/m^2^) was admitted to our referral center for a clinical evaluation due to FD secondary to the *R112H* mutation in the *GLA* gene. The family medical history was positive for Fabry disease: the patient’s mother, who was also affected by the disease, died at the age of 47 due to acute myocardial infarction (AMI) and stroke.

The proband’s medical history revealed disease onset at the age of 13, presenting with proteinuria. At 20 years of age, she developed renal colic in the context of arterial hypertension and hypercholesterolemia. The diagnosis of FD was made in 2006, when a kidney biopsy revealed the typical zebra bodies of Fabry nephropathy. The leukocyte enzyme activity was 2.4 nmol/mg/h (reference values > 3). Soon after, she started enzyme replacement therapy (ERT) with agalsidase beta (1 mg/kg/bw eow) together with ACE inhibitor. Subsequently, due to shortage of agalsidase beta, the treatment was switched to agalsidase alfa (0.2 mg/kg/bw eow).

In 2010, cardiac magnetic resonance imaging (MRI) showed no signs of cardiac involvement. In 2018, because of the worsening of kidney function and proteinuria (creatinine di 1.96 mg/dL, eGFR 33 mL/min, urea 98 mg/dL, serum total proteins 54 g/L, proteinuria 1.56 g/die, albuminemia 28 g/L), a second kidney biopsy was performed: light microscopy revealed diffuse chronic lesions, while electron microscopy showed chronic alterations and lipid inclusions. In 2020, the patient was switched again to agalsidase-beta (1 mg/kg/bw eow). Finally, in 2021 the patient underwent kidney transplantation. In the same period the lyso-Gb3 was 2.3 nmol/L.

In August 2024, the patient underwent further evaluation at our center. Potential multisystem involvement was assessed: no signs of cardiac disease were detected (IVS 7 mm; absence of left ventricular hypertrophy on cardiac MRI); at the peripheral nervous system (PNS) level, no acroparesthesia was noted, though mild hypohidrosis was present; brain MRI revealed millimetric foci of gliosis in the white matter; there were no signs of cornea verticillata; audiometry was within normal limits, and no angiokeratomas were observed. The Lyso-Gb3 levels were 2.91 nmol/mL. Blood tests indicated impaired kidney function, with creatinine at 3.18 mg/dL (eGFR 16 mL/min), leading to the patient being placed on the waiting list for a second kidney transplant.

**Figure 1:**
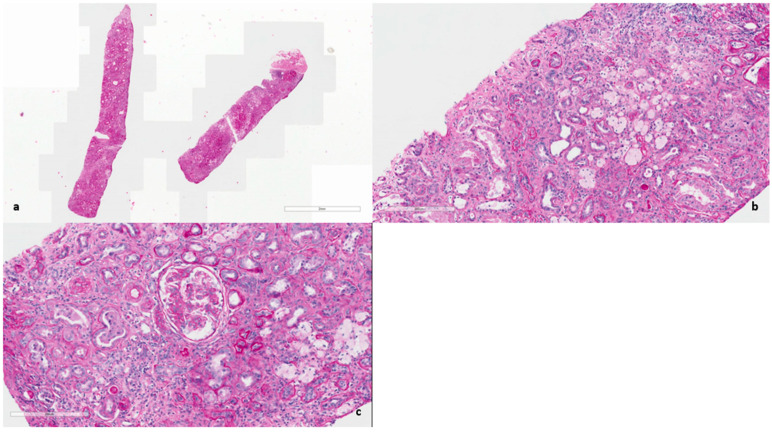
(**a**) Hematoxylin and Eosin (×400). Diffuse interstitial fibrosis and glomerular sclerosis. (**b**) Periodic acid–Schiff (×400). Chronic interstitial nephritis with scattered tubulitis and diffuse foam cells foci. (**c**) Periodic acid–Schiff (×400). Glomerulus nearly globally sclerosed with multiple adhesions. Moderate arteriolosclerosis.

**Figure 2:**
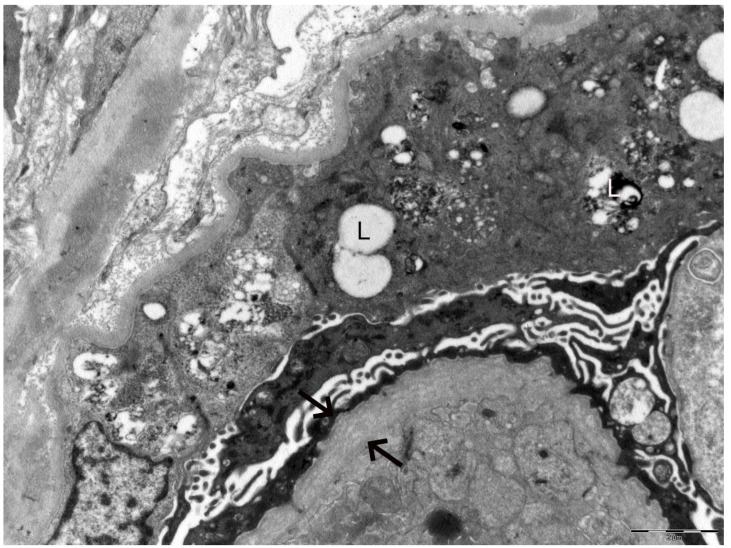
Electron microscopy of the kidney biopsy. The arrows indicate marked remodeling and multilamellation of the glomerular basement membrane. The letter **L** highlights lipid droplets, autophagic vacuoles, and distorted myeloid bodies present within podocytes.

**Figure 3:**
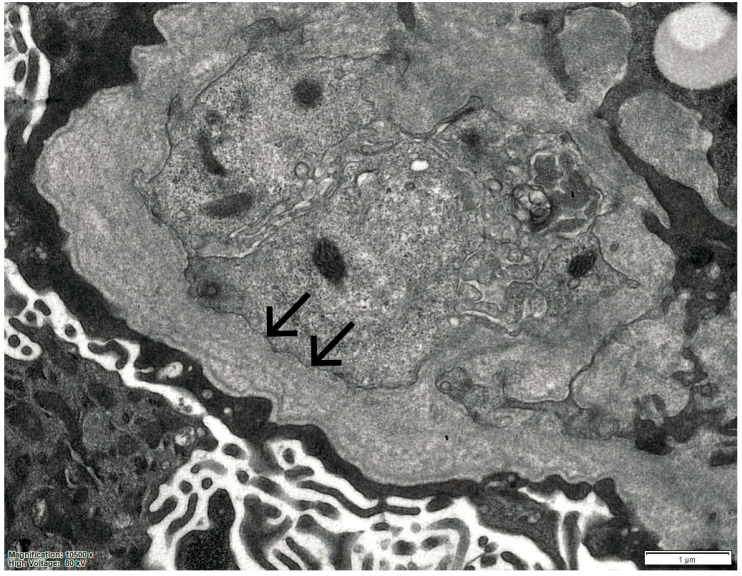
Electron microscopy of the kidney biopsy. The arrows indicate marked remodeling and multilamellation of the glomerular basement membrane.

In 2025, owing to the unusually rapid loss of graft function (creatinine 3.89 mg/dL, eGFR 14 mL/min, urea 102 mg/dL, serum total proteins 56 g/L, AUCR 127 mg/g crea, proteinuria 0.81 g/die), the kidney biopsy performed on the native kidney in 2018 was re-evaluated at our center: a diagnosis of chronic interstitial nephritis with advanced glomerular sclerosis and severe arteriolar nephrosclerosis was established (Figure 1). On re-evaluation of the biopsy by electron microscopy, the residual glomerular capillary loops exhibited marked remodeling and multilamellation of the glomerular basement membrane, consistent with an Alport-like ultrastructural pattern. Podocytes contained abundant lipid droplets, autophagic vacuoles, and distorted myeloid bodies. Remnants of electron-dense deposits and areas of hyaline material were also seen. Numerous interstitial foam cells were present (Figure 2 and Figure 3).

These ultrastructural findings prompted us to perform genetic testing for Alport syndrome, which revealed a heterozygous pathogenic variant, *G912R*, in the *COL4A4* gene.

A timeline summarizing the patient’s clinical course is provided in Table 1.

## 3. Discussion

*R112H* is a missense mutation that results in an amino acid substitution in *GLA*. *R112* is positioned within the loop of the barrel domain in the *GLA* structure, close to the molecule’s surface, and the amino acid substitution does not impact the active site. Patients carrying the *R112H* mutation generally exhibit a mild *GLA* enzymatic activity reduction leading to nearly normal plasma lyso-Gb3 levels [8,9,10,11].

Generally, most patients carrying the R112H mutation are males and exhibit mild symptoms (primarily affecting the kidney system) with a non-classic FD phenotype [4,5,6,7,8,9]. However, some studies have reported severe manifestation, such as ESKD in one male patient at 39 years old [11]. Rombach et al. showed that among eleven patients carrying the *R112H* mutation, ten exhibited no symptoms of classic Fabry disease phenotype, while one male developed kidney insufficiency at the age of 43 [4]. Tanaka et al. reported the case of a male who showed only kidney involvement as well [18]. Sakuraba et al. reported that in 207 Japanese FD patients, five males with *R112H* had a late onset phenotype [9]. Only two studies have reported female patients with the *R112H* variant exhibiting mild isolated proteinuria during adulthood [11,19].

In our study, the patient carrying the R112H mutation exhibited a slightly reduced enzymatic activity (2.4 nmol/mg/h) and mildly elevated plasma lyso-Gb3 levels (2.91 nmol/L). Nevertheless, she showed a severe and early impairment of kidney function, without evidence of cardiac or cerebral involvement, requiring the early start of hemodialysis and subsequent kidney transplantation at the age of 37. The clinical and histopathological findings indicate a rapid progression of Fabry nephropathy in a young female patient with a variant that is generally associated in the literature with late-onset phenotypes [4,9].

Therefore, the presence of significant histological lesions, along with those typical of Fabry nephropathy, and the rapid and early progression to ESKD, rule out the pathogenicity of a late-onset variant in our case.

Of note, we recognized similar histologic lesions as reported in the kidney biopsy of an analogous case report of a female with the *R122H* variant, which initially suggested a possible complement-related disease but was subsequently excluded by genetic testing [11].

Moreover, the re-evaluation of the renal biopsy revealed features suggestive of Alport syndrome, subsequently confirmed by genetic testing, which identified a heterozygous pathogenic variant, *G912R*, in the *COL4A4* gene.

The pathogenic *COL4A4* variant identified in the patient is located within the triple-helical domain of the extracellular matrix protein type IV collagen α4 chain, leading to the substitution of a glycine residue that plays a critical role in maintaining the structural stability of the type IV collagen network.

Considering the early onset of kidney damage in a patient carrying a Fabry mutation typically associated with late-onset disease, it is likely that Alport syndrome may have significantly contributed to the renal decline.

Cases of coexistence of Fabry disease and Alport syndrome are extremely rare and have been only sporadically reported in the literature. Importantly, to date, no case has been documented with definitive confirmation of both diseases by combined genetic and histopathological evidence.

Ren et al. reported a patient with Fabry disease and immunoglobulin A nephropathy presenting with Alport syndrome–like histological findings, underscoring the diagnostic overlap between these entities. The diagnosis of both conditions was based solely on histopathological evaluation: IgA nephropathy was identified by light microscopy and immunofluorescence, whereas Fabry disease was recognized by electron microscopy, which demonstrated sphingolipid accumulation within podocytes and mesangial cells. Genetic testing would be necessary to confirm Fabry disease and to exclude Alport syndrome in light of the glomerular basement membrane alterations [20].

Similarly, Hao et al. reported a case of a patient with IgA nephropathy initially suspected to be associated with Fabry disease based on renal biopsy findings, including the presence of zebra bodies. However, subsequent whole-exome sequencing revealed a *c.3209C > T* mutation in the *COL4A3* gene, confirming Alport syndrome, while no mutation was detected in the *GLA* gene [21].

More recently, Ponleitner et al. documented a male patient with mild renal impairment and microhematuria, in whom whole-exome sequencing revealed novel variants in both the *COL4A4* (*c.1181G > T*, *p.Gly394Val*) and *GLA* (*c.460A > G*, *p.Ile154Val*) genes; however, the clinical and laboratory presentation was more consistent with Alport syndrome, and the pathogenic role of the *GLA* variant remained uncertain [22].

Our case is therefore particularly significant, as it represents the first reported instance with evidence from both renal histopathology and genetic testing, demonstrating the coexistence of Fabry disease and Alport syndrome.

Moreover, this experience should prompt us to investigate potential causes of unexplained findings that cannot be clarified by a simple evaluation of the clinical history.

## 4. Conclusions

In conclusion, the *R112H* mutation in the *GLA* gene exhibits a wide phenotypic spectrum, ranging from asymptomatic individuals to those with severe manifestations predominantly affecting the kidneys.

Our findings support the concept that this variant is associated with a predominantly renal phenotype. However, the distinctive feature of the present case is the unusually rapid progression of renal damage, which led to early renal replacement therapy in a young female patient.

The re-evaluation of the renal biopsy and subsequent genetic testing revealed the coexistence of a heterozygous pathogenic *COL4A4* variant (*G912R*), indicating a concurrent diagnosis of Alport syndrome. This dual genetic condition likely contributed to the early and severe deterioration of renal function, emphasizing the complexity of genotype–phenotype correlations in inherited nephropathies.

This case highlights the crucial importance of an integrated diagnostic approach combining genetic and histopathological investigations, especially in patients with atypical or unexpectedly severe clinical courses. A thorough evaluation of unexplained renal findings may uncover coexisting genetic disorders, leading to a more accurate diagnosis, better risk stratification, and timely therapeutic intervention aimed at slowing disease progression toward end-stage kidney disease.

## Figures and Tables

**Table 1:** Timeline of the patient’s clinical events.

Clinical Timeline	
Age	Clinical Event
13	Onset of disease: proteinuria detected
20	Renal colic episodes, arterial hypertension and hypercholesterolemia
22	Dignosis of Fabry disease confirmed by kidney biopsy (leukocyte *GLA* activity 2.4 nmol/mg/h); Enzyme Replacement Therapy
26	Cardiac MRI showed no signs of cardiac involvement
34	Worsening proteinuria led to a second kidney biopsy: chronic lesions
37	Kidney transplantation; lyso-Gb3 levels: 2.3 nmol/L
40	Clinical and histological comprehensive evaluation; Lyso-Gb3: 2.91 nmol/L

## Data Availability

The original contributions presented in this study are included in the article. Further inquiries can be directed to the corresponding author.

## Abbreviations

The following abbreviations are used in this manuscript:

FD	Fabry disease
GLA	Galactosidase alpha
Gb3	Globotriaosylceramide
Lyso-Gb3	Globotriaosylsphingosine
ESKD	End-stage kidney disease
VUS	Variants of unknown significance
AS	Alport syndrome
GBM	Glomerular basement membrane
XLAS	X-linked Alport syndrome
ADAS	Autosomal dominant Alport syndrome
ARAS	Autosomal recessive Alport syndrome
AMI	Acute myocardial infarction
ERT	Enzyme replacement therapy
ACE	Angiotensin-converting enzyme
MRI	Magnetic resonance imaging
IVS	Interventricular septum
PNS	Peripheral nervous system
eGFR	Estimated glomerular filtration rate
